# Improving Electrolyte Sustainability for Sodium‐Ion Capacitors by Combining a Bio‐Based Solvent With a Low‐Fluorine Salt

**DOI:** 10.1002/cssc.202502493

**Published:** 2026-02-08

**Authors:** Andrea Hainthaler, Manuel J. Pinzón, Maria Arnaiz, Rosalía Cid, Yiyue Lu, Jon Ajuria, Andrea Balducci

**Affiliations:** ^1^ Institute of Technical and Environmental Chemistry Friedrich Schiller University Jena and Center for Energy and Environmental Chemistry (CEEC) Jena Jena Germany; ^2^ Centre for Cooperative Research on Alternative Energies (CIC energiGUNE) Basque Research and Technology Alliance (BRTA) Vitoria‐Gasteiz Spain

**Keywords:** *γ*‐Valerolactone, electrolyte, presodiation, sodium‐ion capacitor, sustainable chemistry

## Abstract

This work focuses on improving the sustainability of electrolytes for sodium‐ion capacitors (SICs). Through the combination of a low‐fluorinated salt, namely sodium difluoro(oxalato)borate (NaDFOB), and the bio‐based solvent *γ*‐Valerolactone (GVL), a new electrolyte formulation (1 mol L^−1^ NaDFOB in GVL) is being studied for application in SICs. Remarkably, the performance of the SIC full‐cells is very comparable to the most commonly used formulation of sodium hexafluorophosphate in ethylene carbonate:propylene carbonate (1 mol L^−1^ NaPF_6_ in EC:PC). Furthermore, presodiation strategies were compared for the novel electrolyte system. The in situ oxidation of a sacrificial salt (sodium squarate, Na_2_C_4_O_4_) incorporated into the positive electrode yielded comparable results to the ex situ electrochemical approach. X‐ray photoelectron spectroscopy studies revealed that depending on the presodiation strategy, the solid‐electrolyte‐interphase composition varies significantly.

## Graphical abstract

This work investigates a novel, sustainable electrolyte for application in sodium‐ion capacitors by combining a low‐fluorine salt, sodium difluoro(oxalato)borate (NaDFOB), with a bio‐based solvent, *γ*‐Valerolactone (GVL). Furthermore, the in situ sacrificial salt (sodium squarate, Na_2_C_4_O_4_) oxidation technique has been studied as a presodiation alternative to reduce the processing steps.

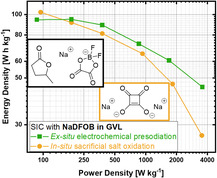

## 1 Introduction

Sodium‐ion capacitors (SICs) are an emerging energy storage technology that offer power density and cycle life comparable to electrochemical double‐layer capacitors (EDLCs), but with higher energy density [1, 2, 3, 4, 5, 6, 7, 8, 9, 10]. These characteristics are achieved by combining a faradic electrode (battery‐type) with a capacitive electrode (EDLC‐type) within the same device. The most commonly employed electrode materials in SICs are Hard Carbon (HC, battery‐type) as negative and Activated Carbon (AC, capacitive type) as positive electrode [1, 4]. These materials are cost‐effective, abundant and eco‐friendly (derived from biowaste) [11]. The electrolyte also plays a major role in the sustainability as well as the performance of a device. In sodium‐ion batteries (SIBs), 1 mol L^−1^ sodium hexafluorophosphate in ethylene carbonate:propylene carbonate (1 M NaPF_6_ in EC:PC) has been established as a standard electrolyte and is also often employed in SICs [10, 12, 13].

When taking a closer look at the electrolyte formulation, it becomes evident that it has a large impact on the environmental footprint of the device. Both EC and PC are solvents based on petro‐industry, largely originating from fossil fuels [14, 15, 16]. Furthermore, the synthesis of NaPF_6_ has a significant impact on resource depletion. Also, the high amounts of fluorine in NaPF_6_ are being debated as a safety risk (HF formation) [17]. For these reasons, the introduction of alternatives for both salts and solvents displaying higher sustainability and lower fluorine content appears to be of great importance for the development of advanced SICs.

A less fluorinated salt, which could be used as replacement for NaPF_6_, is sodium difluoro(oxalato)borate (NaDFOB). NaDFOB is a very stable salt, generating no toxic or dangerous products when exposed to air or water [18]. Precisely, it has been shown that, under the same conditions, NaPF_6_ forms HF when in contact with water, while no HF is formed with NaDFOB [18]. Furthermore, a 1 M solution of the salt in PC proved to be stable >5 V versus Na^+^/Na, indicating good oxidative stability [18]. When NaDFOB is oxidized, it has been proposed that oligomers and polymers are formed as well as NaF, all of which contribute to the formation of a stabilizing passivation layer [19, 20, 21]. NaDFOB has already been used in SIBs before, especially as additive enhancing both their safety and performance [22, 23, 24, 25, 26, 27, 28]. Furthermore, it has been employed in sodium‐metal batteries (SMBs) due to its excellent stabilization of sodium metal [27, 29, 30].

As mentioned before, the commonly employed solvents, EC and PC, can be viewed critically from a sustainability perspective. Thus, a bio‐based solvent will be introduced to the SIC technology: *γ*‐Valerolactone (GVL). This solvent can be produced from biomass via acid‐catalyzed conversion of sugars and subsequent hydrogenation or directly in a one‐pot synthesis from fructose [31, 32]. The solvent has also been shown to be biodegradable [31]. Furthermore, GVL displays a wide liquid range (−31°C–207°C), high flash point (96°C), and relatively low density (1.05 g mL^−1^), making it interesting for electrochemical energy storage devices [33]. In the last years, our group investigated the use of this bio‐based solvent in EDLCs, lithium‐ion batteries (LIBs) as well as lithium‐ion capacitors (LICs) showing that its use allows for the realization of high performance devices [34, 35, 36, 37]. However, to the best of our knowledge, GVL has not yet been employed in SICs.

In this work, we aim to develop a novel electrolyte for SICs displaying higher sustainability and lower fluorine content with respect to the state‐of‐the‐art. We therefore consider the combined use of NaDFOB and GVL. Specifically, we investigate the use of an electrolytic solution containing 1 M NaDFOB in GVL. Initially, the chemical‐physical properties of this electrolyte, as well as its application in combination with HC and AC electrodes, are considered in detail. Afterwards, lab‐scale SICs are assembled and tested to understand the impact of the electrolyte on the performance of these devices. Finally, a comparison of different presodiation strategies is made: The commonly employed ex situ electrochemical approach is compared to an in situ sacrificial salt oxidation (sodium squarate, Na_2_C_4_O_4_). A special focus is laid on the resulting solid‐electrolyte‐interphase (SEI) layers varying depending on the choice of presodiation technique.

## 2 Experimental Section

### 2.1 Electrode Preparation

Electrodes were prepared following a previously reported procedure [38].

For hard carbon (HC) and activated carbon (AC) electrodes, a mass ratio of 90 wt% active material (AM) to 5 wt% binder to 5 wt% conducting agent was chosen. Independent of the used active material, Na‐Carboxymethyl cellulose (Na‐CMC, CRT2000 GA, Walocel) was used as binder and carbon black (C‐NERGY Super C45, Imerys) as conducting agent. The AC (YP‐50F) and HC (Biocarbotron‐5 μm) were purchased from Kuraray. All the solids were weighed in and mixed with ca. three parts deionized water. The mixture was processed for 3 min at 50 oscillations per second in a ball mill. Using the doctor‐blade technique, films of 100 μm (for HC) and 150 μm (for AC) were manufactured onto an Aluminum foil current collector. After drying overnight at room temperature, electrodes with a surface area of 1.13 cm^2^ were punched. The electrodes were dried at 0.5 mbar at 60°C overnight and then inserted into an argon‐filled glovebox (Mbraun, O_2_ and H_2_O < 1 ppm). The mass loading of the electrodes containing HC was ≈1.9 mg cm^−2^, while the ones containing AC was ≈2.8 mg cm^−2^.

For the sacrificial salt containing AC‐electrodes (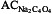), the slurry was formulated using a composition of 60 wt% AC (YP‐50F, Kuraray), 30 wt% Na_2_C_4_O_4_ as sacrificial salt, 5 wt% conductive carbon (C‐NERGY Super C65, Imerys), and 5 wt% polyvinylidene fluoride (PVdF) binder in *N*‐methyl‐2‐pyrrolidone (NMP) solvent. A two‐step mixing protocol was employed: First, Na_2_C_4_O_4_ was ball‐milled together with C65 to ensure carbon coating and particle size reduction. This blend was then dispersed into a previously prepared PVdF solution. Finally, the AC was added, and the mixture was homogenized under high shear. The slurry was then cast onto etched Aluminum current collector within a specific wet thickness in order to obtain ≈3.5 mg cm^−2^ mass loadings.

When AC electrodes were tested in half‐cells, oversized AC electrodes were used as the counter electrodes (CEs). A mass ratio of AM 90 wt%, conducting agent 5 wt%, and binder 5 wt% was chosen. 100 mg of AC YP‐50F (Kuraray), 5.6 mg of carbon black, and 9.3 mg of polytetrafluoroethylene (PTFE, 60 wt% in H_2_O, Sigma Aldrich) were weighed in and suspended in ethanol (EtOH). Whilst stirring, EtOH was steadily added to form a viscous material. The slurry was rolled out to a thin film with a glass bar and folded back up again multiple times. When needed to maintain the smoothness of the film, a few drops of EtOH were added. Finally, the mixture was rolled out to a thin film, and electrodes with a diameter of 12 mm were punched. The electrodes were dried overnight at 60°C and 0.1 mbar. The mass loading of the electrodes was ≈20 mg cm^−2^.

### 2.2 Electrolyte Preparation

The solvent *γ*‐Valerolactone (GVL), as well as the employed conductive salt sodium difluoro(oxalato)borate (NaDFOB), were both purchased from Sigma Aldrich. The initial water content of GVL was reduced to ≤20 ppm by filtering over dried aluminum oxide (90 active basic, Merck) under argon atmosphere. The water content was determined via Karl‐Fischer titration (C20 Coulometric KF Titrator, METTLER TOLEDO). The electrolyte 1 M NaDFOB in GVL was prepared in‐house in an argon‐filled glovebox. The electrolyte 1 M NaPF_6_ in EC:PC (1:1, wt) was provided by E‐lyte (<10 ppm water content) and used without further purification. In the following, these electrolytes will be indicated as NaDFOB in GVL and NaPF_6_ in EC:PC.

### 2.3 Electrolyte Characterization

The viscosity was measured in the temperature range between −10 and 80°C at an Anton‐Paar MCR 102 rotational viscometer, applying a shear rate of 1000 s^−1^. For conductivity measurements, 0.5 mL of electrolyte was added into a sealed glass cell with two parallel platinized platinum electrodes having a known cell constant (ca*.* 1 cm^−1^, examined with KCl solution). The cell was placed into a climate chamber (BINDER) for electrochemical impedance measurement (amplitude: 5 mV, frequency range: 100 mHz–100 MHz) using a ModulabXM ECS potentiostat (Solartron) to obtain the alternating current resistance and calculate the electrolyte conductivity.

### 2.4 Assembly of Half‐Cells: Anodic Stability, HC Half‐Cells, Al Dissolution, and AC Half‐Cells

Swagelok cells were assembled in an argon‐filled glovebox. As a separator, a glass fiber disk (Whatman GF/D) soaked with 150 μL of electrolytic solution was used. For all measurements, either a BioLogic VMP‐3 or a BioLogic MPG‐2 potentiostat were utilized.

For determining the anodic stability of the electrolyte, a platinum disc was employed as working electrode (WE), an oversized AC electrode as CE and sodium metal as reference electrode (RE). A linear sweep measurement at 1 mV s^−1^ was performed, and a threshold of 100 µA cm^−2^ was selected as oxidative stability limit.

For the investigation of the HC‐electrodes, metallic sodium (Sigma Aldrich) was used as CE and RE (three‐electrode set‐up). In galvanostatic cycling with potential limitations (GCPL)‐measurements, the performance was tested between 5 mV and 2 V versus Na^+^/Na. Ten cycles at 50 mA g^−1^ were conducted to get differential capacity plots. For that purpose, the raw data (potential and capacity) was processed to be monotonous. Then, the data was reduced, and the remaining data points were made equidistant. After the ten cycles at 50 mA g^−1^, cycling tests were performed at 1 A g^−1^. Separate cells were built to investigate the rate performance. Hereby, the cells were activated by ten cyclic voltammetry (CV) cycles at 0.1 mV s^−1^, followed by five charge/discharge cycles at 50, 100, 200, 500, 1000, 2000, 3000, and 50 mA g^−1^.

The anodic dissolution of the Aluminum current collector was investigated utilizing half‐cells containing Al as WE and Na as CE and RE. The Al was charged with 0.5 mV s^−1^ up to 4.5 V versus Na^+^/Na and the potential was held for 3 h. Afterwards, the electrode was discharged to 2 V versus Na^+^/Na. The procedure was repeated ten times.

For the investigations of the AC electrodes, an oversized AC electrode was used as CE and sodium metal as RE. CV measurements to determine the oxidative cut‐off potential were performed at 1 mV s^−1^. Five cycles were conducted at each cut‐off potential (4.0, 4.1, 4.2, 4.3, and 4.4 V vs. Na^+^/Na). The Coulombic efficiencies were determined by integrating the oxidative/reductive area beneath the graph and dividing the reductive integration area by the oxidative one. The oxidative stability was also investigated employing GCPL: 50 cycles at each cut‐off potential (4.0, 4.1, 4.2, 4.3, 4.4, 4.5, 4.6, and 4.0 V vs. Na^+^/Na) were conducted at 500 mA g^−1^. The rate‐capability of AC half‐cells was tested within a potential range of 2–4 V versus Na^+^/Na for current rates of 0.1, 0.2, 0.5, 1, 2, 5, 10, and 20 A g^−1^ for ten cycles each. The floating stability was tested at 4.2 V for 200 h (capacitance determined at 200 mA g^−1^ between 2 and 4 V vs. Na^+^/Na).

### 2.5 Assembly of Half‐Cells: Pretreatment Before SIC Assembly

As presodiation step, a HC electrode was precycled in a separate half‐cell (three‐electrode set‐up, Na as CE and RE), before assembling a SIC.

The electrode was discharged at 18.8 mA g^−1^ for 3 h, followed by a discharge of 37.2 mA g^−1^ until 5 mV versus Na^+^/Na were reached. The potential of 5 mV versus Na^+^/Na was held for 1 h. Subsequently, the HC was charged up to 2.5 V versus Na^+^/Na at 37.2 mA g^−1^ and discharged to 5 mV versus Na^+^/Na at 9.3 mA g^−1^. The HC was then charged to 2 V versus Na^+^/Na by 37.2 mA g^−1^ and discharged at the same rate. Another five cycles of charge/discharge at 37.2 mA g^−1^ followed. The final potential of 5 mV versus Na^+^/Na was held until ≤30% of 37.2 mA g^−1^ of current was flowing, which took around 2 h. Normally, the HC potential upon disconnection was between 50 and 150 mV versus Na^+^/Na, depending on the IR‐drop and the timing. The cell was disassembled in the glovebox, and the presodiated HC‐electrode was inserted into the SIC device.

### 2.6 Assembly of SIC Full‐Cells

#### 2.6.1 Ex Situ Electrochemical Presodiation

In a three‐electrode set‐up, the presodiated HC was combined with an AC electrode and metallic sodium. Before starting the SIC measurement, the uncycled AC electrode needed to be charged to realize a broad cell voltage window. For that purpose, the AC electrode was in situ connected to the sodium metal as CE/RE. The AC was then brought to 4.0 V versus Na^+^/Na, using current densities of 250 or 500 mA g^−1^. The potential was held for 30 min. Then, the AC‐electrode was connected as WE (positive electrode), the HC‐electrode as CE (negative electrode), and Na as RE, and the SIC device was tested galvanostatically between 1 V < *E*_cell_ < 3.8 V. Current densities of 25, 50, 100, 250, 500, 1000, and 25 mA g^−1^ were applied. Note that the current rate was normalized on the sum of both electrodes’ active material masses. After the rate test, cycling at 200 mA g^−1^ for 150 cycles followed. To further determine the stability, separate cells for float measurements have been evaluated. First, ten activation cycles at 0.1 A g^−1^ were conducted, followed by a resting time of 10 h. In a loop five cycles at 0.1 A g^−1^, 5 h of float at *E*_cell_ = 3.8 V, and 5 h of resting time were applied for 24 times resulting in a total floating time of 120 h.

#### 2.6.2 In Situ Sacrificial Salt Oxidation

In a three‐electrode set‐up, the sacrificial salt containing ${\mathrm{AC}}_{{\mathrm{Na}}_{2}C_{4}O_{4}}$ was combined with a pristine HC as negative electrode and metallic sodium as RE. The cell was cycled galvanostatically between 2 V < *E*_cell_ < 4 V at a rate of $25\;\mathrm{mA}\;{g^{-1}}_{{\mathrm{Na}}_{2}C_{4}O_{4}}$ (normalized on the mass of sacrificial salt) for 10 cycles. This ensured the complete oxidation of the sacrificial salt—responsible for the SEI formation—before testing the rate‐capability. Here, current densities of 25, 50, 100, 250, 500, 1000, and 25 mA g^−1^ were applied between 1 V < *E*_cell_ < 3.8 V (current normalized on the sum of both electrodes’ active material masses). Afterwards, cycling tests were performed at 200 mA g^−1^ for 150 cycles within the same cell voltage range.

### 2.7 Surface Chemical Characterization by X‐Ray Photoelectron Spectroscopy (XPS)

After presodiation, each type of negative electrode was removed from its respective cell inside a glovebox under inert argon atmosphere and subjected to a washing protocol to eliminate residual salts and separator fragments. The protocol comprised three 1 mL applications of *γ*‐Valerolactone (GVL) delivered as moderate‐velocity jets at 15 s intervals. Once the solvent had evaporated, electrodes were transferred directly from the glovebox to the spectrometer load‐lock using a hermetically sealed transfer tool to preserve the SEI. XPS was conducted on a Phoibos 150 spectrometer (SPECS Surface Nano Analysis) in an ultrahigh‐vacuum chamber (≈5 × 10^−10^ mbar) equipped with nonmonochromatic Al K*α* (*h* *ν*  = 1486.61 eV) and Mg K*α* (*h* *ν* = 1253.64 eV) sources. Spectra were acquired in fixed analyzer transmission (FAT) mode with a 400 µm spot. Survey spectra used a 0.5 eV energy step and 90 eV pass energy; high‐resolution spectra used a 0.1 eV step and 30 eV pass energy. Data was processed with CASA XPS (v2.3.26). The adventitious hydrocarbon C 1*s* peak at 284.8 eV was used to calibrate the binding‐energy scale, with further refinement using additional reference peaks. Spectral envelopes were fitted with Lorentzian‐asymmetric line shapes and a Shirley background.

## 3 Results

### 3.1 Physicochemical Properties and Anodic Stability

To realize high‐performance SICs, the electrolyte must display suitable transport properties and a large electrochemical stability window. Table 1 compares the dynamic viscosity, ionic conductivity, and anodic stability of 1 M NaDFOB in GVL with those of the commonly employed 1 M NaPF_6_ in EC:PC. All the values were recorded at room temperature (20°C). It can be seen that NaPF_6_ in EC:PC displays higher conductivity (7.81 vs. 5.12 mS cm^−1^) and slightly higher anodic stability values than the novel electrolyte (5.0 vs. 4.8 V vs. Na^+^/Na). NaDFOB in GVL, however, yielded a lower viscosity (4.68 vs. 5.37 mPa s^−1^). Considering these values, NaDFOB in GVL certainly appears to have properties suitable for application in SICs.

**TABLE 1:** Physicochemical properties and anodic stability of NaPF_6_ in EC:PC versus NaDFOB in GVL. Dynamic viscosity, ionic conductivity, and anodic stability values recorded at 20°C.

	Dyn. Viscosity, mPa s^−1^	Ionic conductivity, mS cm^−1^	Anodic stability, V vs. Na^+^/Na
1M NaPF_6_ in EC:PC	5.37 [38]	7.81 [38]	5.0
1M NaDFOB in GVL	4.68	5.12	4.8

### 3.2 HC Negative Electrodes

**FIGURE 1:**
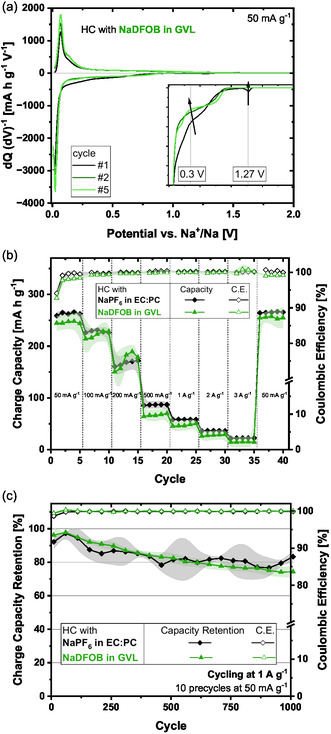
Electrochemical results of HC half‐cells. (a) Differential capacity with respective electrolyte NaDFOB in GVL, (b) comparison between NaDFOB in GVL and NaPF_6_ in EC:PC rate‐test, and (c) cycling test.

The novel electrolyte was first tested with the battery‐type electrode HC within a potential range of 5 mV–2 V versus Na^+^/Na. The differential capacity analysis of the first cycle shows a distinct reduction peak at ca. 1.3 V versus Na^+^/Na, which has been ascribed to the decomposition of NaDFOB [39] and/or to the reduction of impurities, namely oxalate esters (Figure 1a) [40]. At 0.3 V versus Na^+^/Na, a broad signal is obtained, probably originating from the GVL decomposition. Both of these peaks disappear or decrease in the following cycles, indicating less decomposition. The initial Coulombic efficiency (ICE) of the electrode was 70%, which is a value comparable to that observed for the conventional electrolyte NaPF_6_ in EC:PC. As can be seen in the rate‐test results (Figure 1b), these first formation cycles are sufficient to form a stable SEI layer enabling capacities of ≈250 mA h g^−1^ at 50 mA g^−1^ and ≈50 mA h g^−1^ at 1 A g^−1^. The results also show that the capacity values are comparable to the state‐of‐the‐art electrolyte. For SIC application, the longevity of the device is crucial. Thus, the stability of HC electrodes in NaDFOB in GVL was tested, carrying out charge–discharge at 1 A g^−1^. As shown, after more than 600 cycles, the electrodes are able to retain 80% of their maximum capacity, and at the end of the cycling process (1000 cycles), they retained more than 70% of their maximum capacity (Figure 1c). In comparison, the conventional electrolyte, NaPF_6_ in EC:PC, displays an increase in capacity within the first 50 cycles. Afterwards, the capacity fades, resulting in ≈80% of the maximum capacity after 1000 cycles.

Overall, the novel electrolyte NaDFOB in GVL is compatible with the HC electrode material. Even without the oftentimes employed EC, a stable SEI is formed within the first cycles, allowing for comparable performances to the state‐of‐the‐art (NaPF_6_ in EC:PC) electrolyte in terms of capacity.

### 3.3 AC Positive Electrode and Aluminum Stability

**FIGURE 2:**
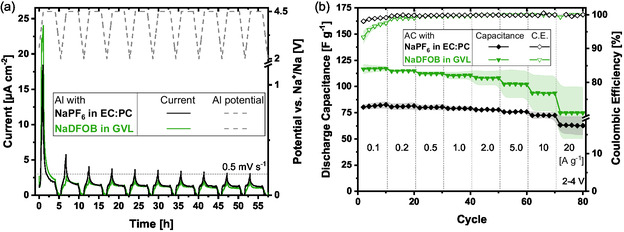
Electrochemical results of Al and AC half‐cells. (a) Anodic dissolution test of Al half‐cell with NaDFOB in GVL versus NaPF_6_ in EC:PC at 4.5 V versus Na^+^/Na and (b) rate‐test of AC half‐cell with NaDFOB in GVL versus NaPF_6_ in EC:PC within a potential range of 2–4 V versus Na^+^/Na.

Before investigating the positive electrode active material, the anodic stability of the current collector was tested. Therefore, an Aluminum half‐cell with NaDFOB in GVL was assembled, cycled between 2 and 4.5 V (with a potential hold step at 4.5 V), and the resulting current was monitored. Figure 2a shows that a clear current peak can be observed in the first cycle, while the following cycles did not yield significant current amounts (<3 µA cm^−2^). It can thus be concluded that within the first cycle, a passivation layer is formed on top of the Al current collector, protecting the material from further reactions in the subsequent cycles. This is in line with previous reports on NaDFOB being a potent additive for Al passivation [20, 25, 41, 42]. As a reference, the same measurement was performed for the conventional NaPF_6_ in EC:PC electrolyte, showing similar behavior (Figure 2a).

To find the optimum potential window for the use of AC electrodes in NaDFOB in GVL, CV measurements with increasing upper cut‐off potential were performed (Figure S1a). For 4 V versus Na^+^/Na, a Coulombic efficiency of 98.4% resulted in the 4th cycle. At higher potentials, the Coulombic efficiency decreases significantly. For example, at 4.2 V versus Na^+^/Na, only a value of 93.8% could be achieved. It is important to note that above 4.0 V versus Na^+^/Na the applied current density affects the Coulombic efficiency of the charge–discharge process substantially: When the electrodes are cycled for example at 4.2 V versus Na^+^/Na at 500 mA g^−1^, they display Coulombic efficiencies greater than 98% (Figure S1b). It can thus be concluded that above 4 V versus Na^+^/Na Faradic reactions are thermodynamically possible and will occur if a low enough rate is applied, therefore harming the performance of the cell. Hence, a potential window of 2–4 V versus Na^+^/Na was chosen for further investigations.

After the optimal potential window was identified, a rate‐test was conducted cycling the AC electrodes between 2 and 4 V versus Na^+^/Na. Under these operating conditions, they deliver 110 F g^1^ at 1 A g^−1^ and show excellent rate capability up to 5 A g^−1^ (Figure 2b). A comparison with the conventional electrolyte was added as well. The novel electrolyte enables higher capacitance values throughout all applied current densities. The very different electrolyte composition probably leads to a chemically different interphase apparently allowing for better charge transport. For investigating the stability of the AC electrodes with the novel electrolyte, the electrodes were floated at 4.2 V versus Na^+^/Na for 200 h showing no capacitance loss after that time (Figure S1c).

All in all, the use of NaDFOB in GVL in combination with AC electrodes appears very promising. Even though the current collector is well protected at very high potentials (minimum of 4.5 V versus Na^+^/Na), the maximum stable potential for AC electrodes within this electrolyte seems to be 4 V versus Na^+^/Na. For this work it was therefore decided to stay within this stable region.

### 3.4 SIC Full‐Cells

**FIGURE 3:**
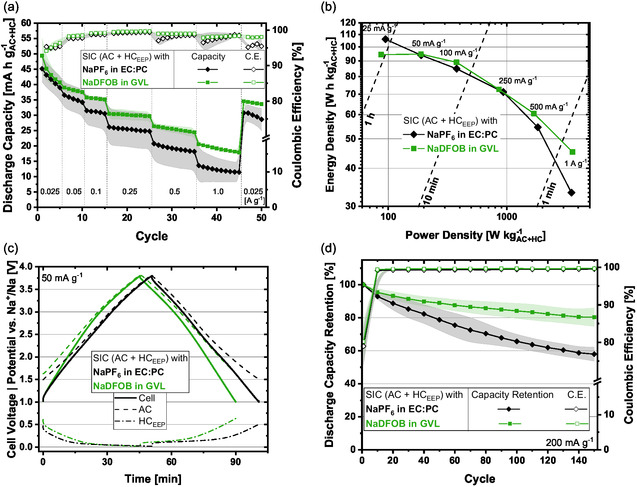
Electrochemical results for SIC (AC + HC_EEP_) full‐cells. A pristine AC and an ex situ electrochemically presodiated HC (HC_EEP_) were combined with the investigated electrolyte NaDFOB in GVL, tested within a voltage range of 1 V < *E*_cell_ < 3.8 V and compared with the state‐of‐the‐art electrolyte NaPF_6_ in EC:PC. (a) Rate‐test, (b) Ragone‐plot, (c) potential and voltage profiles at 50 mA g^−1^, and (d) cycling stability at 200 mA g^−1^.

SIC full‐cells were assembled containing an ex situ electrochemically presodiated HC electrode (denoted HC_EEP_, for details see Section 2) and a pristine AC electrode [43]. The active mass balancing AC:HC was 1.5:1 (for details about the capacity balancing, cf. Table S1 in the Supporting Information). A rate performance test was conducted to evaluate the functionality and especially the high‐power capabilities of the device with the novel electrolyte NaDFOB in GVL (Figure 3a). ICEs of 93% and capacity values of ≈40 mA h g^−1^ within the first rate of 25 mA g^−1^ were obtained (capacities and currents are normalized on the sum of both active material masses). The capacity values are decreasing in the first few cycles due to activation processes. At higher rates (1 A g^−1^), still high values of capacity are retained (≈20 mA h g^−1^). In the last five cycles, again at 25 mA g^−1^, it can be seen that the system has stabilized, yielding a capacity of 35 mA h g^−1^. In comparison, the conventional electrolyte NaPF_6_ in EC:PC, shows lower capacity values as well as lower Coulombic efficiencies throughout all applied rates (Figure 3a).

A comparison of the respective best performing SIC cell containing NaDFOB in GVL versus NaPF_6_ in EC:PC is drawn: The energy and power densities of the investigated devices in a Ragone‐plot reveal enhanced high‐rate performance for the system employing the novel electrolyte (Figure 3b). For example, at 0.5 A g^−1^ the device containing NaDFOB in GVL displays an energy density of 60 W h kg^−1^, while that of the device containing the conventional electrolyte is 50 W h kg^−1^. At a current density of 50 mA g^−1^, the performance output of the two different electrolytes is very comparable. Figure 3c shows the corresponding potential and voltage profiles displaying slightly higher charge/discharge times for the conventional electrolyte. For both electrolytes, the electrode potentials move within stable regions, i.e., 20 mV < *E*_HC_ < 600 mV and 1.5 V < *E*_AC_ < 3.8 V versus Na^+^/Na. It is worth noticing that for the device containing NaDFOB in GVL, the charge/discharge process at 1 A g^−1^ takes ≈1 min, and that under these operating conditions it delivers an energy density of >40 W h kg^−1^ and power density of >3500 W kg^−1^ (Figure 3b). These values are very promising when compared to other lab‐scale devices [4].

For investigating the stability of the NaDFOB in GVL containing full‐cells, cycling experiments were conducted, yielding >80% capacity retention after 150 cycles at 200 mA g^−1^ (Figure 3d). Except for the very first cycle, Coulombic efficiencies of >99.6% were obtained throughout the experiment. The potential excursion of both electrodes remained stable as well (AC: 2.2–3.8 V and HC: 1.4–0.03 V vs. Na^+^/Na, cf. Figure S2a). The conventional electrolyte, NaPF_6_ in EC:PC, only retains ≈60% of its initial capacity under the same conditions (Figure 3d). Thus, the novel electrolyte formulation increases the cycle life of SIC full‐cells.

To further investigate the system's stability, float tests have been performed at a cell voltage of 3.8 V and room temperature (Figure S2b). The SIC containing NaDFOB in GVL retains ≈30% of its initial capacity after 100 h of floating, which is slightly worse to the performance of the state‐of‐the‐art electrolyte (≈40% capacity retention) [38].

### 3.5 Alternative Presodiation Strategy: In Situ Oxidation of Sacrificial Salt

So far, an ex situ electrochemical presodiation technique has been applied. To increase the safety, efficiency, and cost‐effectiveness of the full‐cell production, an alternative approach has been investigated [44]: The in situ oxidation of a sacrificial salt incorporated into the positive electrode, releasing sodium cations upon oxidation [43]. The choice was made for sodium squarate (Na_2_C_4_O_4_), whose lithium analog has already been shown to be applicable to LICs [45, 46] and which has been employed for SICs [44, 47]. During its oxidation, Na_2_C_4_O_4_ releases sodium ions, CO, CO_2_, and carbon deposits [47].

**FIGURE 4:**
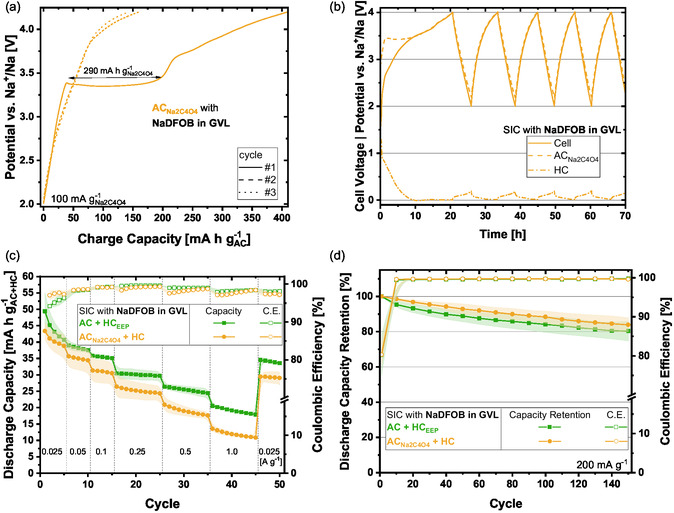
Electrochemical results of AC containing sacrificial salt (${\mathrm{AC}}_{{\mathrm{Na}}_{2}C_{4}O_{4}}$) and corresponding SIC with NaDFOB in GVL. (a) ${\mathrm{AC}}_{{\mathrm{Na}}_{2}C_{4}O_{4}}$ half‐cell capacity with salt oxidation plateau, (b) SIC (${\mathrm{AC}}_{{\mathrm{Na}}_{2}C_{4}O_{4}}$ + HC) potential and voltage profiles, (c) SIC rate‐test comparison between different presodiation strategies (AC + HC_EEP_ and ${\mathrm{AC}}_{{\mathrm{Na}}_{2}C_{4}O_{4}}$ + HC), and (d) comparison of SIC cycling results at 200 mA g^−1^.

Electrodes containing 60 wt% AC and 30 wt% Na_2_C_4_O_4_ (furtherly denoted as ${\mathrm{AC}}_{{\mathrm{Na}}_{2}C_{4}O_{4}}$) were fabricated (all details are reported in Section 2) and investigated for their applicability in the studied system. First, ${\mathrm{AC}}_{{\mathrm{Na}}_{2}C_{4}O_{4}}$ half‐cells were tested to gain insight into the oxidation of the salt and the associated released capacity. Figure S3 shows the potential profile of the ${\mathrm{AC}}_{{\mathrm{Na}}_{2}C_{4}O_{4}}$ electrodes when cycled at $100\;\mathrm{mA}\;{g^{-1}}_{{\mathrm{Na}}_{2}C_{4}O_{4}}$ (normalized on the mass of sacrificial salt) between 2 and 4.2 V versus Na^+^/Na. A clear oxidation plateau at ≈3.4 V versus Na^+^/Na is visible in the first cycle. This value is lower than those already reported (≈3.8 V vs. Na^+^/Na) which could be due to other employed electrolytes or slightly different active materials [46]. Most importantly, the oxidation plateau is only prominent in the first cycle. Afterwards, the expected capacitive, triangular potential profiles result. When the capacity of the charge process is plotted (Figure 4a) an irreversible capacity of $290\;\mathrm{mA}\;h\;{g^{-1}}_{{\mathrm{Na}}_{2}C_{4}O_{4}}$ is obtained, which is in good practical agreement with the theoretical maximum capacity of $339\;\mathrm{mA}\;h\;{g^{-1}}_{{\mathrm{Na}}_{2}C_{4}O_{4}}$ [46].

Subsequently, SICs were built‐up containing ${\mathrm{AC}}_{{\mathrm{Na}}_{2}C_{4}O_{4}}$ as positive electrode and pristine HC as negative electrode. The active mass balancing between AC:HC was equal to 1.55:1 (which is a value comparable to the previously utilized AC:HC 1.5:1). Ten cycles of $25\;\mathrm{mA}\;{g^{-1}}_{{\mathrm{Na}}_{2}C_{4}O_{4}}$ between 2 V < *E*_cell_ < 4 V were conducted to ensure the full oxidation of the sacrificial salt. As can be seen from Figure 4b, in the first cycle the AC reaches ≈4 V versus Na^+^/Na and the HC reaches a minimum of 4 mV versus Na^+^/Na. Thus, a good mass balancing was chosen seeing as the potential of the positive electrode does not exceed the electrolyte stability and on the negative electrode side potentials above 0 V versus Na^+^/Na are obtained. In this first cycle low Coulombic efficiencies of ≈25% result due to the irreversible oxidation of the sacrificial salt. Afterwards, the rate‐capability of the SIC was tested between 1 V < *E*_cell_ < 3.8 V. Figure 4c compares the performance of the sacrificial salt containing SIC with the previously shown SIC (realized with ex situ electrochemically presodiated HC, denoted as HC_EEP_). Comparable initial capacity values of ≈40 mA h g^−1^ were obtained. At higher rates, the HC_EEP_ system performs better both in terms of capacity and Coulombic efficiency. The slow rate at the end of the rate‐test proves that both systems are stable and can output approximately the same amount of capacity (≈30 vs. 35 mA h g^−1^ at 25 mA g^−1^). Afterwards, cycling tests were conducted which yielded again comparable results of ≈80% capacity retention for both presodiation methods after 150 cycles at 200 mA g^−1^ (Figure 4d). Interestingly, the in situ sacrificial salt oxidation presodiation technique leads to slightly higher capacity retention values.

### 3.6 XPS Comparison of Different Presodiation Strategies

**FIGURE 5:**
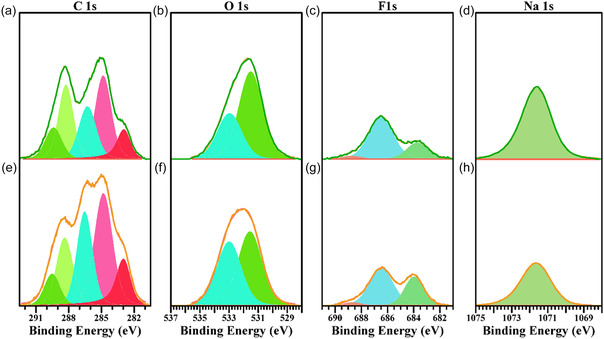
XPS deconvoluted high resolution spectra for desodiated electrodes after presodiation. (a) C 1*s*, (b) O 1*s*, (c) F 1*s*, and (d) Na 1*s* obtained from ex situ electrochemically presodiated electrodes. (e) C 1*s*, (f) O 1*s*, (g) F 1*s*, and (h) Na 1*s* obtained from electrodes presodiated by in situ oxidation of sacrificial salt.

The impact of the different presodiation strategies on the surface of the HC electrodes was investigated. Figure 5 presents the high‐resolution XPS spectra of desodiated electrodes after being subjected to each presodiation protocol: ex situ electrochemical presodiation (Figure 5a–d), and in situ oxidation of sacrificial salt (Figure 5e–h; for comparison to the pristine HC electrode, cf. Figure S4). In both types of negative electrodes, the Na 1*s* peak appears at 1071.6 eV and is attributed to the main organic (Na—C—O, Na—C=O) and inorganic (NaF) constituents of the SEI. The C 1*s* region displays the characteristic asymmetric C=C signal from the HC bulk, shifted to lower binding energies (282.9 eV) because of the potential gradient induced by SEI formation [48]. A distinct peak at 284.8 eV corresponds to C—C/C—H bonding, while additional features at 286.5, 288.3, and 289.4 eV are assigned to C—O—C, O—C=O, and —(O=C)—O—(C=O)— species, respectively, representing the organic component of the SEI. This interpretation is further supported by the O 1*s* spectra, which exhibit peaks at 532.9 eV (O—C—O) and 531.5 eV (O—C=O). The F 1*s* region shows two main contributions: a peak at 686.5 eV associated with NaDFOB decomposition products and surface residues, and another at 683.8 eV corresponding to NaF.

The comparison of SEI component ratios across both electrode types indicates that organic species with O—C=O and —(O=C)—O—(C=O)— motifs, arising from electrolyte decomposition, are present in similar amounts for the two presodiation routes. In contrast, C—O—C products and the C—C bonding signal are more abundant for the in situ sodiated electrode, consistent with effective incorporation of oxidation products from the sacrificial salt and a correspondingly more robust SEI. Furthermore, the NaF signal for in situ sodiation is approximately twice that for ex situ sodiation, supporting a more highly structured SEI in the in situ case and likely underpinning the superior capacity retention.

## 4 Conclusion

An alternative electrolyte for SIC application has been investigated. The combination of a low‐fluorinated salt sodium difluoro(oxalato)borate in the bio‐based solvent *γ*‐Valerolactone (1 M NaDFOB in GVL) has been shown to be suitable for the state‐of‐the‐art SIC consisting of HC and AC. The novel electrolyte is capable of protecting the Al current collector at high potentials (4.5 V versus Na^+^/Na) as well as forming a stable SEI layer on the HC surface. It yields comparable capacity and capacitance, respectively, values to the state‐of‐the‐art electrolyte NaPF_6_ in EC:PC for both half‐cell investigations. The performance of the SIC full‐cell is very comparable as well, whereby the novel electrolyte even outperforms the conventional electrolyte at high rates. It was thus shown that a simplified (one less component) and greener (less fluorine; solvent production bio‐based) electrolyte yields comparable performance values to the state‐of‐the‐art electrolyte. To further increase the sustainability of the device, an alternative presodiation strategy has been employed: The use of sodium squarate (Na_2_C_4_O_4_) allowed for one less processing step in the fabrication of a full‐cell and yielded comparable results to the ex situ electrochemical (conventional) presodiation approach. Additionally, the use of Na_2_C_4_O_4_ promotes a more robust, inorganic‐rich SEI that is less prone to redissolution, thereby improving capacity retention.

## Supporting Information

Additional supporting information can be found online in the Supporting Information section. **Supporting Fig. S1:** Electrochemical results of Activated Carbon (AC) half‐cells with NaDFOB in GVL. Investigation of the upper cut‐off potential via CV (a) and via GCPL (b). Floating test at 4.2 V (c). **Supporting Fig. S2:** Electrochemical results for SIC (AC + HC_EEP_) full‐cells. Potential profiles during long‐term cycling (a) and float results at E_cell_ = 3.8 V compared between NaDFOB in GVL and NaPF_6_ in EC:PC (b). **Supporting Fig. S3:** Electrochemical results for AC containing sacrificial salt Na_2_C_4_O_4_. Potential profiles between 2‐4.2 V vs. Na^+^/Na displaying the salt oxidation plateau at ≈3.4 vs. Na^+^/Na. **Supporting Fig. S4:** XPS deconvoluted high resolution spectra for pristine hard carbon electrode. Na 1s (left), O 1s (center) and C 1s (right). **Supporting Table S1:** Capacity and mass balancing between HC and AC with NaDFOB in GVL for different rates.

## Author Contributions

**Andrea Hainthaler:** investigation (lead), writing – original draft (lead), writing – review & editing (supporting). **Manuel J. Pinzón:** investigation (supporting). **Maria Arnaiz:** investigation (supporting), writing – original draft (supporting), writing review & editing (supporting). **Rosalía Cid:** investigation (supporting), writing – review & editing (supporting). **Yiyue Lu:** investigation (supporting). **Jon Ajuria:** funding acquisition (equal), writing – review & editing (supporting). **Andrea Balducci:** funding acquisition (lead), writing – review & editing (lead).

## Funding

This work was supported by HORIZON EUROPE Reforming and enhancing the European Research and Innovation system (101092080), and Bundesministerium für Bildung und Forschung (03XP0533D).

## Conflicts of Interest

The authors declare no conflicts of interest.

## Supporting information

Supplementary Material

## Data Availability

The data that support the findings of this study are available from the corresponding author upon reasonable request.
