# Supplementary material for: Improving Electrolyte Sustainability for Sodium‐Ion Capacitors by Combining a Bio‐Based Solvent With a Low‐Fluorine Salt
Source: ChemSusChem. 2026 Feb 8;19(3):e202502493. doi: 10.1002/cssc.202502493 (PMC12883094; doi:10.1002/cssc.202502493)
Supplement: Supplementary file 1 — Supplementary Material [file CSSC-19-e202502493-s001.pdf]

SUPPORTING INFORMATION

Improving Electrolyte Sustainability for Sodium-ion Capacitors by Combining a Bio-based Solvent with a Low-fluorine Salt

Andrea Hainthaler[1], Manuel J. Pinzón[2], Maria Arnaiz[2], Rosalía Cid[2], Yiyue Lu[1], Jon Ajuria[2], Andrea Balducci[1*]

[1] Institute of Technical and Environmental Chemistry, Friedrich Schiller University Jena and Center for Energy and Environmental Chemistry (CEEC) Jena, Philosophenweg 7a, 07743 Jena, Germany

[2] Centre for Cooperative Research on Alternative Energies (CIC energiGUNE), Basque Research and Technology Alliance (BRTA), Alava Technology Park, Albert Einstein 48, 01510 Vitoria-Gasteiz, Spain

Determination of Energy and Power Density

For determining the (gravimetric) energy density E_g of the SIC devices the product of current and voltage is integrated over the time and divided by the mass (Equation 1). This equation is used for non-ideal EDLCs as well as hybrid systems and was thus chosen for this work.

$$E_g = \frac{1}{m} \int V(t)\, I(t)\, \mathrm{d}t \qquad (1)$$

The maximum specific power density is obtained by using the same power calculation, hence, the product of voltage and current. Then, the negative maximum of the power of one GCD cycle is determined. With this technique the power after the IR-drop is determined leading to the maximum available power.

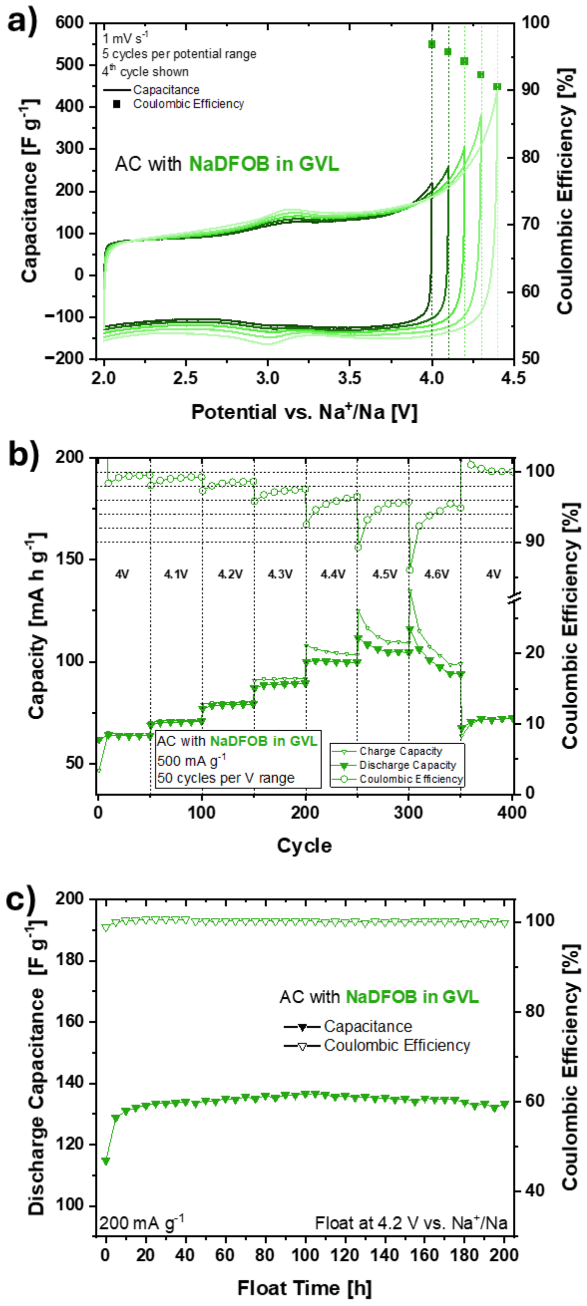

Figure S1. Electrochemical results of Activated Carbon (AC) half-cells with NaDFOB in GVL. *Investigation of the upper cut-off potential via CV (a) and via GCPL (b). Floating test at 4.2 V (c).*

Table S1. Capacity and mass balancing between HC and AC with NaDFOB in GVL for different rates.

Current Rate [A g^{-1}]	Capacity balancing (q_{HC} /	Mass balancing (m_{HC} / m_{AC})

	q_{AC})	
0.1	3.60	0.8
0.2	3.13	0.8
0.5	1.27	0.8
1	1.01	0.8
2	0.74	0.8

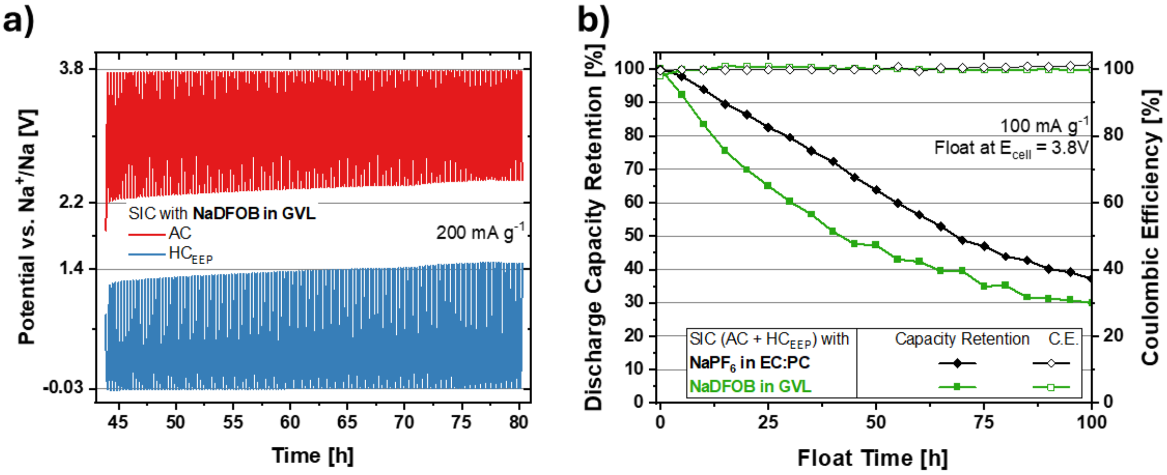

Figure S2. Electrochemical results for SIC (AC + HC_{EEP}) full-cells. *Potential profiles during long-term cycling (a) and float results at E_{cell} = 3.8 V compared between NaDFOB in GVL and $NaPF_6$ in EC:PC (b).*

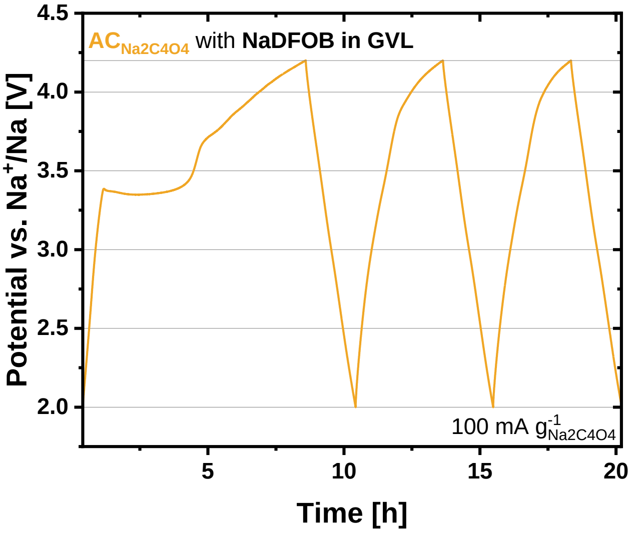

Figure S3. Electrochemical results for AC containing sacrificial salt $Na_2C_4O_4$. *Potential profiles between 2-4.2 V vs. Na^+/Na displaying the salt oxidation plateau at ~ 3.4 vs. Na^+/Na.*

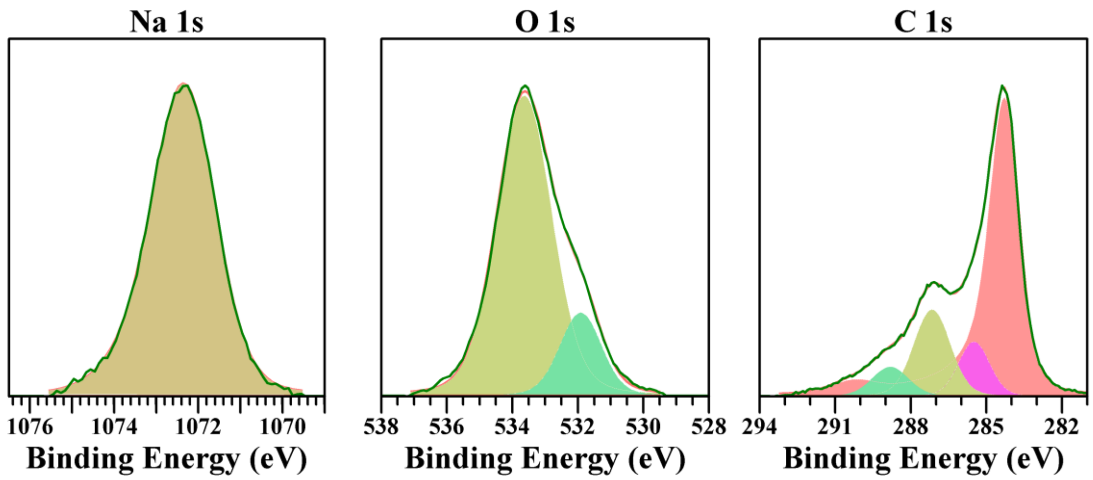

Figure S4. XPS deconvoluted high resolution spectra for pristine hard carbon electrode. *Na 1s (left), O 1s (center) and C 1s (right).*

Fig.S4 presents the high-resolution spectra obtained from a pristine hard carbon-based electrode. The Na 1s peak (1072.3 eV) is attributed to sodium in the CMC. In the O 1s region, two peaks are observed: one at 533.6 eV corresponding to C-O bonds, and another at 531.9 eV attributed to C=O bonds. The C=O peak is linked to the binder, while the C-O peak results from contributions of these bonds in both the binder and functional groups on the hard carbon surface. The deconvolution of the C 1s region reveals a prominent asymmetric peak at 284.2 eV, characteristic of the C=C bond in hard carbon, along with a peak at 285.5 eV associated with C-O functionalities and C-C bonds on the surface of the hard carbon. Additional peaks at 287.1 eV and 288.8 eV correspond to the C-O and C=O bonds identified in the O 1s region, both of which are associated with the CMC binder.
